# The Effect of Time Resolution on Apparent Transition Path Times Observed in Single-Molecule Studies of Biomolecules

**DOI:** 10.1021/acs.jpcb.2c05550

**Published:** 2022-10-04

**Authors:** Dmitrii E. Makarov, Alexander Berezhkovskii, Gilad Haran, Eli Pollak

**Affiliations:** ^†^Depatment of Chemistry and ^‡^Oden Institute for Computational Engineering and Sciences, University of Texas at Austin, Austin, Texas78712, United States; § Eunice Kennedy Shriver National Institute of Child Health and Human Development, National Institutes of Health, Bethesda, Maryland20892, United States; ∥ Department of Chemical and Biological Physics, Weizmann Institute of Science, Rehovot76100, Israel

## Abstract

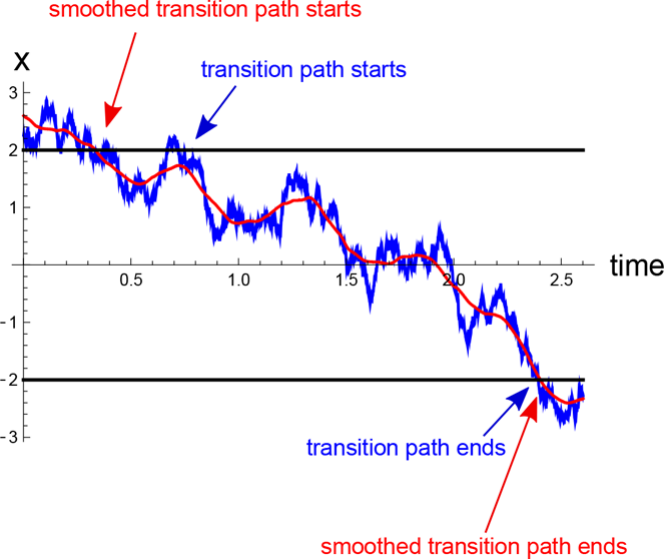

Single-molecule experiments have now achieved a time resolution allowing observation of transition paths, the brief trajectory segments where the molecule undergoing an unfolding or folding transition enters the energetically or entropically unfavorable barrier region from the folded/unfolded side and exits to the unfolded/folded side, thereby completing the transition. This resolution, however, is yet insufficient to identify the precise entrance/exit events that mark the beginning and the end of a transition path: the nature of the diffusive dynamics is such that a molecular trajectory will recross the boundary between the barrier region and the folded/unfolded state, multiple times, at a time scale much shorter than that of the typical experimental resolution. Here we use theory and Brownian dynamics simulations to show that, as a result of such recrossings, the apparent transition path times are generally longer than the true ones. We quantify this effect using a simple model where the observed dynamics is a moving average of the true dynamics and discuss experimental implications of our results.

## 1 Introduction

The past few years have seen impressive advances in the experimental monitoring of biomolecular dynamics, especially in relation to folding and unfolding transitions in proteins and nucleic acids. A new and exciting development has been the capability to measure folding/unfolding transition paths, that is, the trajectories that biomolecules take as they cross free energy barriers between folded and unfolded states. The detailed measurement of transition paths as a function of time, which can only be carried out on individual molecules, has started to provide us with an intimate view of what is really happening to biomolecules as they undergo conformational rearrangements.^1,2^ While the initial studies of transition paths focused on the average transition path times,^3,4^ more recent work investigated finer details such as transition path time distributions,^5−8^ which have been shown to be particularly informative about the folding dynamics,^9−11^ as well as transition path shapes and velocities.^12,13^

Several experimental techniques have been applied in studies of transition paths. Force spectroscopy,^1^ particularly using optical tweezers, has shown significant promise. The technique, as applied particularly by Woodside and co-workers, involves tethering a biomolecule to two beads that are trapped under tension in optical traps, and the displacement of the beads—and thus the extension of the molecule as it moves—is recorded. The unfolded molecule incurs larger extensions than the folded one, so that monitoring the extension as a function of time provides direct information on the state of the molecule. Provided that the time resolution of the optical tweezers is high enough, one may follow a biomolecule as it transits between folded and unfolded states.

A second method, pioneered by Eaton and co-workers, employs Fluorescence Resonance Energy Transfer (FRET) between two fluorophores attached to a biomolecule to probe its transitions.^2^ The donor fluorophore is photoexcited, and depending on its distance from the acceptor fluorophore, it may transfer its energy to the latter, which would then emit the light. Single-molecule FRET experiments allow one to monitor the FRET efficiency as a function of time, revealing the time dependence of the distance between the two fluorophores. One can then monitor how the interfluorophore distance evolves when the folding barrier is crossed—that is, observe transition paths.

Recently, a third technique has been introduced to study transition paths. It involves monitoring an electric current blockade in a nanopore as a biomolecule passes through it; a folded molecule blocks the current more than an unfolded molecule. Gruebele, Wanunu, and co-workers have recently reported such measurements of transition paths with a time resolution of 0.5 μs (ref (14)).

Each of these methods has its pros and cons. For example, a central question in the optical tweezers experiment involves unraveling the filtering effect due to the motion of the sluggish beads.^15−18^ In the single-molecule FRET experiments, on the other hand, it is challenging to obtain a high-enough photon flux in order to follow closely the relatively fast folding/unfolding transitions. Finally, monitoring the electric current blockade in a nanopore involves an effect of the pore on the transition. A key point—which is the topic of the present paper—is that none of the experimental methods truly measures the instantaneous value of the experimental observable reporting on the molecule’s dynamics (such as the molecular extension or the donor–acceptor distance). Rather, the observed trajectory is always time-averaged/smoothed. The natural question, then, is what are the implications of such time averaging when interpreting single-molecule trajectories and gleaning from them information on the observed conformational transitions?

A related question is how to interpret the measured transition paths theoretically. Often, experimental results are compared to approximate analytical results obtained using simple potentials (see, e.g., refs (3 and 5)) rather than solving the diffusion equation numerically (see, e.g., refs (6 and 7)). In such approximate solutions, one often replaces the absorbing boundary conditions required to obtain the correct transition path times (see next Section) with open boundary conditions.^19−23^ But the mean transition path time determined from the diffusion equation with open boundary conditions will, in principle, be longer than the same time determined with absorbing boundary conditions. This observation has recently led to a renewed interpretation of published optical tweezers experiments,^5^ indicating the existence of a long-lived intermediate along the transition path, which was missed due to the employment of open boundary conditions.^24^ Open boundary conditions will include path segments in which the system crosses the boundary but then returns to it, paralleling experimental measurements with limited time resolution. In a sense, trajectory smoothing, which is inherent to experimental studies, blurs the difference between open and absorbing boundary conditions; it is thus not immediately clear which theoretical description is more appropriate to describe experimental data.

There is yet another related question that must be addressed. In any calculation, whether via a numerical solution of the diffusion equation or a more sophisticated molecular dynamics simulation, the resulting numerical data must be time-binned so as to analyze and extract information from the measurements. How much time-averaging is needed? What does this imply for the statistics of the resulting binned trajectories? Are they diffusive, or do they include memory and ballistic effects?

These questions have motivated this paper. We explore them using a simple model cusp-shaped barrier potential, which has the advantage of being tractable analytically, with the results having a clear physical meaning. When analytical results are unavailable, we supplement theory with simulations, particularly to study the effect of smoothing on the apparent properties of transition paths. The rest of this paper is organized as follows. Section 2 explores the effect of the boundary conditions through analytical theory. In Section 3 we study the effect of smoothing on the apparent properties of transition paths. Section 4 concludes with a discussion of the practical implications of our observations for the analysis of experimental distributions of transition-path times in folding-unfolding kinetics of biomolecules.

## 2 Effect of Boundary Recrossings on the Observed Transition Path Time

Consider dynamics along a coordinate *x* (representing the experimental observable) in a bistable potential of mean force *U*(*x*), with the left and the right minima representing the “reactant” and the “product” of a “reaction”, for example, folded and unfolded states of a protein. Transition paths are segments of trajectories *x*(*t*) that stay continuously within a specified transition region, (*a*, *b*), having entered it from one boundary *a*(*b*) and exited through the other, *b*(*a*).

**Figure 1:**
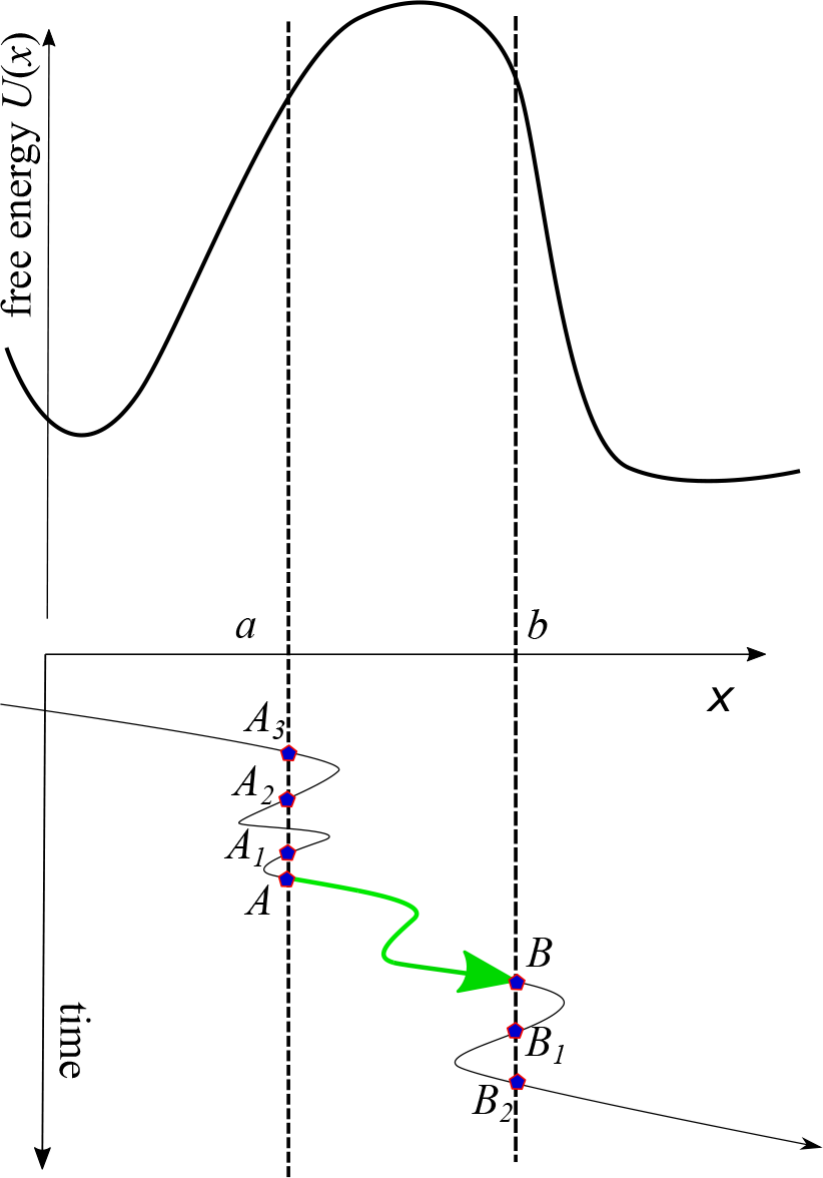
A transition path (green) stays continuously in the transition region (*a*, *b*), entering it by crossing one boundary (here at point A) and exiting through the other (point B). If the trajectory exits and reenters the transition region on a time scale that is shorter than the experimental time resolution, then the entrance point A may be misidentified as, e.g., points A_1_, A_2_, or A_3_, and, likewise, the exit point B may be misidentified as B_1_ or B_2_. This generally increases the apparent transition path time.

An example of a transition path is shown in Figure 1, in green. It enters the transition region through its left boundary *a* at a point denoted A and exits this region at a point B. Given the stochasticity of the trajectory *x*(*t*), it may recross the left boundary multiple times (illustrated in Figure 1 as points A_1_, A_2_, and A_3_) before committing to the transition path shown in green. The segment of *x*(*t*) between A_3_ and A consists of failed transition attempts or “loops”.^25^ Likewise, the exit from the transition region may be followed by multiple reentrance events through the boundary *b* (loops) before the trajectory is finally committed to the “product” potential well.

Current experimental techniques usually do not have sufficient time or positional resolution to precisely pinpoint the last crossing A of the boundary *a* and the first crossing B of the boundary *b*. Indeed, a typical time scale for recrossings *τ*_r_ should be on the order of the velocity autocorrelation time. For a polystyrene bead with a diameter of 2*r* = 1 μm (a typical bead size in the optical tweezers setup), for example, this time is readily estimated using Stokes’ law: , where *m* is the bead mass and η is the water viscosity. This gives *τ*_r_ ≈ 50 ns, at least an order of magnitude shorter than a typical time resolution in such measurements. In FRET measurements, the time resolution is determined by the rate of photon emission: if multiple recrossings occur between the arrival moments of two successive photons, they cannot be detected. In practice, the problem is even more acute than described; several photons are needed in order to define a FRET efficiency value or a distance derived from it with reasonable confidence,^26^ and the exact number may depend on various molecular and photophysical parameters.

What is the effect of misidentifying the precise time where the transition path enters/exits the transition region on the apparent values of the transition path time? Since the point A in Figure 1, where the transition path starts, corresponds to the last time that the left boundary is crossed before the transition path begins, the *measured* time when the transition path starts at the boundary *a* will likely occur before the true time. Likewise, the apparent transition path would end later than the true moment when the boundary *b* is first crossed, thereby terminating the transition path. In other words, the apparent transition path time would include a contribution from the loops, and thus it would be longer than the true transition path time.

To make this argument more quantitative, we need an estimate of the temporal duration of the loop part of the trajectory. To explain how this can be done, we start with describing the standard way of calculating the distribution of the transition path times (and its mean or higher moments) for the case where the dynamics obey the Smoluchowski equation.
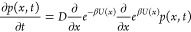
1Here *D* is the diffusivity (which we will assume to be position-independent),  is the inverse thermal energy, and *p*(*x*, *t*) is the probability density of finding the system at point *x* at time *t*. Equivalently, the stochastic time evolution of trajectories *x*(*t*) is described by the overdamped Langevin equation
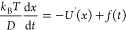
2where *f*(*t*) is a Gaussian-distributed, delta-correlated random force with zero mean, which obeys the fluctuation–dissipation theorem
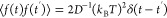
3To obtain the distribution of the transition path times, *p*_TP_(*t*|*a* → *b*), we imagine a trajectory that has just crossed the boundary *a* and is located at *x*_0_ = *a* + ϵ at *t* = 0, where the limit ϵ → 0 will eventually be taken. We follow this trajectory until it either exits the interval (*a*, *b*) through the boundary *a* (in which case it does not belong to the ensemble of transition paths) or through boundary *b* (in which case it is a transition path whose temporal duration contributes to the distribution of the transition path time *p*_TP_(*t*|*a* → *b*)). This distribution is then proportional to the flux exiting the boundary *b*
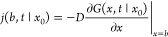
4where *G*(*x*, *t*|*x*_0_) is the Green’s function, which is the solution of eq 1 with the initial condition
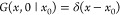
5and absorbing boundary conditions
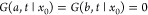
6The absorbing boundary condition at *x* = *a* eliminates the trajectories that fail to make it to the boundary *b*, and thus are not transition paths. Since only a fraction ϕ(*x*_0_ → *b*) of the trajectories succeed in making it to *b*, the distribution of transition path times is obtained from eq 4 by normalizing the flux with the fraction of successful transition paths
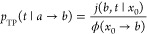
7and finally taking the limit *x*_0_ = *a* + ϵ → *a*.

The “splitting probability” ϕ(*x*_0_ → *b*) can be obtained by integrating the flux *j*(*b*, *t*|*x*_0_)
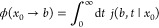
8

Physically, the absorbing boundary condition at *x* = *a* eliminates the contribution from the loops. In contrast, the *first passage* time distribution *p*_FP_(*t*|*a* → *b*) from *x* = *a* to *b* will contain contributions both from transition paths and from trajectories that start at *a* and return to *a* multiple times before arriving at *b* (e.g., pieces of trajectories starting at A_1_, A_2_, and A_3_ in Figure 1). This distribution is obtained by considering all trajectories that start at *x* = *a* and cross the boundary *b* at a later time *t*, regardless of whether or not they exited the interval (*a*, *b*). As such trajectories include both transition paths and loops, the difference between the distributions *p*_FP_(*t*|*a* → *b*) and *p*_TP_(*t*|*a* → *b*) and, particularly, their means, informs us about the contribution from the loops.

**Figure 2:**
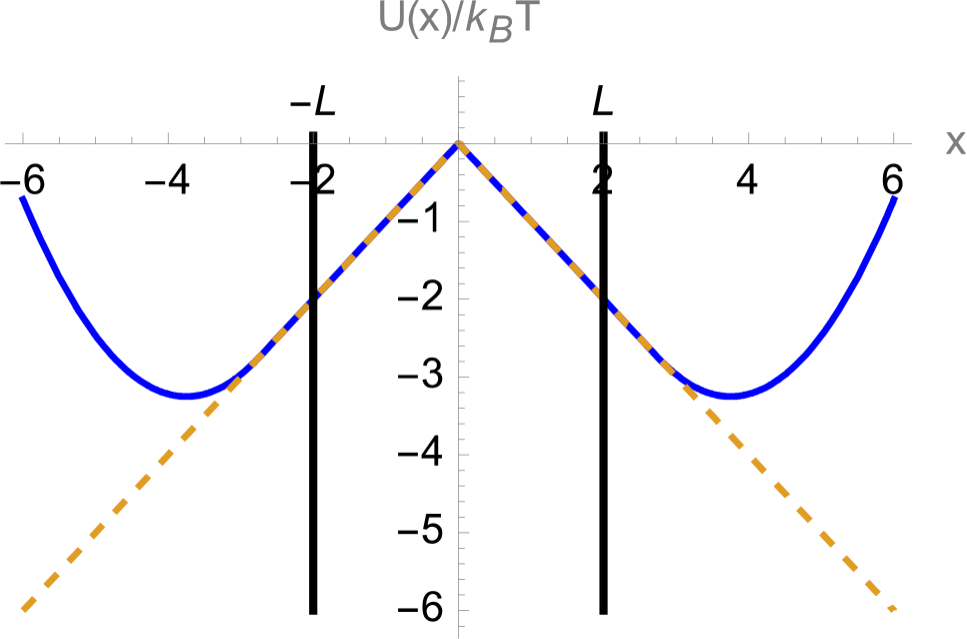
Model double-well potential used here is described by a piecewise function  = , which has a cusp-shaped barrier. Here *x*_1_ = 2.75, *k* = 1, and *F* = 1. The transition region (−*L*, *L*) is indicated by the vertical lines. Conditional first passage times were calculated using the modified potential (eq 9), which is identical to *U*(*x*) in the transition region but lacks potential wells (dashed line). The same potential was used to calculate transition path times using open boundary conditions.

Unlike the distribution of transition path times, the distribution of first passage times *p*_FP_(*t*|*a* → *b*) depends not only on the potential shape inside the transition region (*a*, *b*) but also on the potential outside it. In fact, for a double-well potential (Figure 1) *p*_FP_(*t*|*a* → *b*) will include contributions from trajectories that return to the vicinity of the left potential minimum before eventually crossing the barrier to the right. For a potential well that is deep enough, such events have a much longer time scale (comparable to the inverse of the interwell transition rate) than the loops that we are interested in. Moreover, such events would be easy to resolve experimentally. To exclude such long excursions, we consider the dynamics in a modified potential *Ũ*(*x*), which is identical to *U*(*x*) for *a* < *x* < *b* but which lacks potential wells and has the property that *Ũ*(*x*) → −∞ for *x* → ±∞. More specifically, below we focus on a cusp-shaped potential barrier of the form (Figure 2)
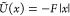
9because dynamics on such a potential is tractable analytically.

We chose the boundaries *a* = −*L*, *b* = +*L* to be located symmetrically with respect to the barrier top *x* = 0. For the potential *Ũ*(*x*), some fraction of the trajectories starting at *a* will escape to the left, never crossing the barrier and reaching the point *b*. We thus define *p*_FP_^(*c*)^(*t*|*a* → *b*) as the distribution of the *conditional* first passage time to reach *b*, provided that it happens. This can be computed in a manner similar to eq 7: We solve eq 1 with the absorbing boundary condition at *b* but not at *a*
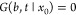
10and then compute the flux (eq 4) of trajectories crossing *b*. Now we have
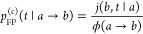
11where ϕ(*a* → *b*) = ∫_0_^∞^dt *j*(*b*, *t*|*a*) is the probability that a trajectory that starts at *x* = *a* reaches the point *b* rather than escapes to −∞ (splitting probability).

**Figure 3:**
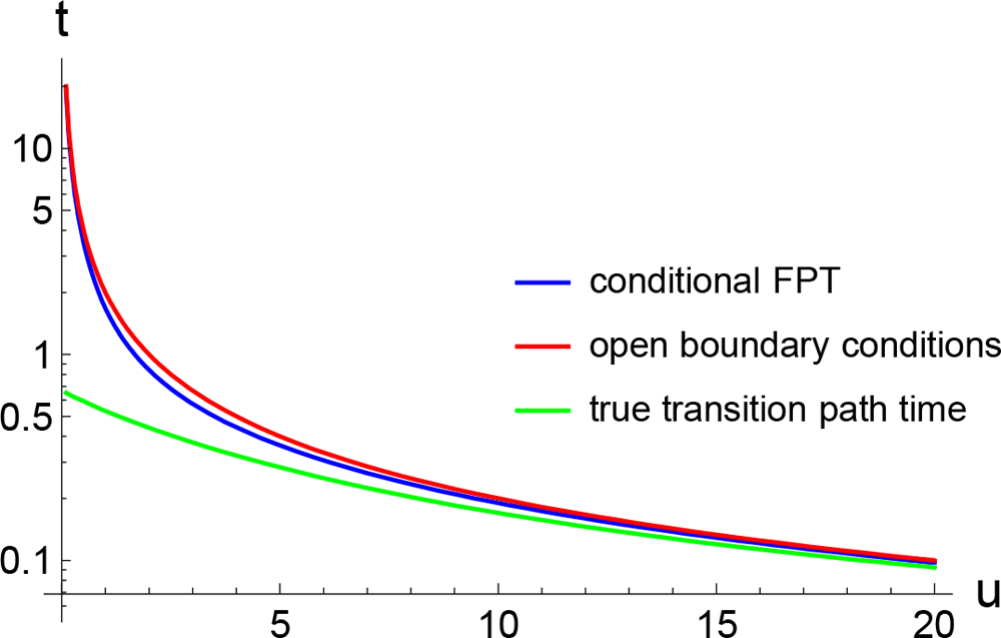
Mean conditional first passage time ⟨*t*_FP_^(*c*)^⟩ (blue line, eq 12), mean transition path time ⟨*t*_TP_⟩_open_ evaluated using open boundary conditions (red line, eq 17), and the true transition path time ⟨*t*_TP_⟩ (green line, eq 15) plotted as a function of the transition path barrier height measured in thermal energy units (eq 14) for the cusp-shaped potential, eq 9. Time is reported in dimensionless units of , where 2*L* is the barrier width (Figure 2) and *D* is the diffusivity. Note the logarithmic time axis.

If every trajectory crossing the boundary *a* were to proceed to *b* without recrossing *a*, the two distributions, *p*_FP_^(*c*)^(*t*|*a* → *b*) and *p*_TP_(*t*|*a* → *b*), would be identical. Physically, if the potential at *x* = *a* is steep enough, any trajectory that recrosses the boundary *a* will, within a negligibly short time, evolve toward *x* = −∞ rather than reenter the transition region. Likewise, a trajectory that has reached point *b* starting from *a* will proceed toward *x* → ∞. Thus, we anticipate that *p*_FP_^(*c*)^(*t*|*a* → *b*) will approach *p*_TP_(*t*|*a* → *b*) as either the force *F* is increased or the transition region width *b* – *a* = 2*L* increases while keeping the force *F* constant. In particular, the mean conditional first passage time
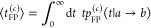
12will approach the mean transition-path time
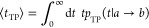
13This is indeed what is observed in this “steep potential” limit, when we calculate these times for the cusp-shaped potential of eq 9 (Figure 3). In this case the distributions *p*_TP_(*t*|*a* → *b*) and *p*_FP_^(*c*)^(*t*|*a* → *b*) only depend on the dimensionless parameter
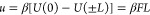
14which is the transition path barrier height^22^ (equal to the barrier measured relative to *x* = ±*L*) normalized by thermal energy. In particular, for this potential we have^27^
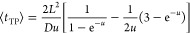
15The explicit expression for ⟨*t*_FP_^(*c*)^⟩ is rather long; how it was calculated is explained in Appendix A.

The no-recrossing assumption is also invoked in the “open boundary conditions” approximation, which is often employed to obtain analytic results for transition path time distributions^19−22^ as well as to fit experimental data.^5^ This approximation is based on eqs 4 and 7 but with the Green’s function *G*(*x*, *t*|*x*_0_) satisfying the absorbing boundary conditions (eq 6) replaced by the Green’s function *G*_open_(*x*, *t*|*x*_0_) satisfying eqs 1 and 5 without the absorbing boundaries. Correspondingly, the splitting probability ϕ(*x*_0_ → *b*) in eq 7 is now replaced by the integral of the flux, which guarantees proper normalization of the estimated distribution of the transition path time.
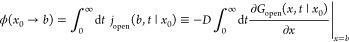
16For the cusp-shaped potential *Ũ*(*x*), the mean transition path time estimated using the open boundary conditions is given by a physically appealing formula (Appendix B)
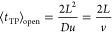
17where *v* = *FDβ* is the mean drift velocity of the system in the presence of a constant force *F*.

We note that the conditional times ⟨*t*_TP_⟩_open_ and ⟨*t*_FP_^(*c*)^⟩, while both affected by loops, are different quantities, and the difference between the two is not merely a change in boundary conditions. As seen in Figure 3, for low (reduced) barrier heights, both of these times are much longer than the mean transition path time, highlighting the significant contribution from trajectories re-entering the transition region. As the barrier height increases, the three times converge to the same value.

**Figure 4:**
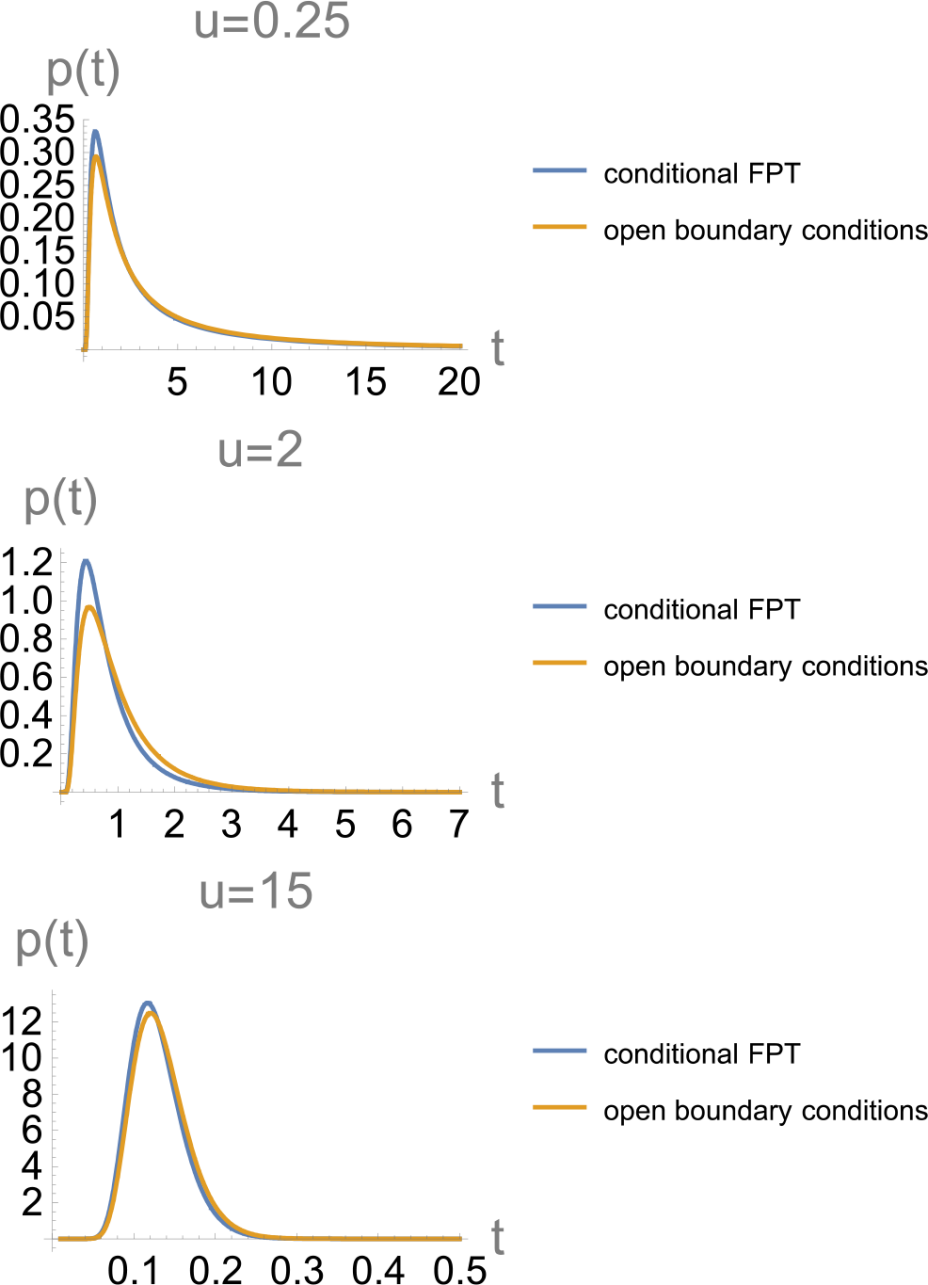
Probability densities of the conditional first passage time (obtained by numerically inverting the Laplace transform of eqs A16–A19) and the transition path time evaluated using open boundary conditions (eq B2) plotted for different values of the transition path barrier height measured in thermal energy units (eq 14) for the cusp-shaped potential, eq 9. Time is reported in dimensionless units of , where 2*L* is the barrier width (Figure 2) and *D* is the diffusivity.

Interestingly, ⟨*t*_TP_⟩_open_ is close to the mean conditional first passage time ⟨*t*_FP_^(*c*)^⟩ at all values of the dimensionless free energy barrier *u*, asymptotically behaving in the same way in the limits *u* → 0 and *u* → ∞ (Figure 3). Moreover, the distributions of the conditional first passage time *p*_FP_^(*c*)^(*t*|*a* → *b*) and of the transition path time *p*_TP_(*t*|*a* → *b*) estimated using open boundary conditions are close to one another for any barrier height (as illustrated in Figure 4). Thus, recrossings of the boundaries of the transition region lengthen both ⟨*t*_TP_⟩_open_ and ⟨*t*_FP_^(*c*)^⟩, as compared to the true transition path time ⟨*t*_TP_⟩, by roughly the same amount, with all three times approaching the same limit at high barriers; specifically, this limit is
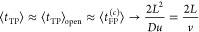
18

## 3 Effect of Trajectory Smoothing on the Apparent Transition Path Time

To understand more quantitatively how temporal resolution of the measurement affects the apparent transition path times, here we adopt a model in which the observed values *x̃* of the quantity of interest differ from the instantaneous values *x*: specifically, the observed trajectory *x̃*(*t*) is a smoothed version of the true trajectory *x*(*t*) obtained by performing a moving average over a certain time window Δ*t*.
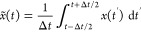
19We note that such smoothing is explicitly used in force spectroscopy studies (see, e.g., ref (5)) to eliminate noise. We perform Langevin dynamics simulations with the full potential *U*(*x*) of Figure 2 and the boundaries *a* = −*L*, *b* = +*L*. We compute smoothed trajectories *x̃*(*t*) and use them instead of *x*(*t*) to analyze the (apparent) transition path ensemble.

**Figure 5:**
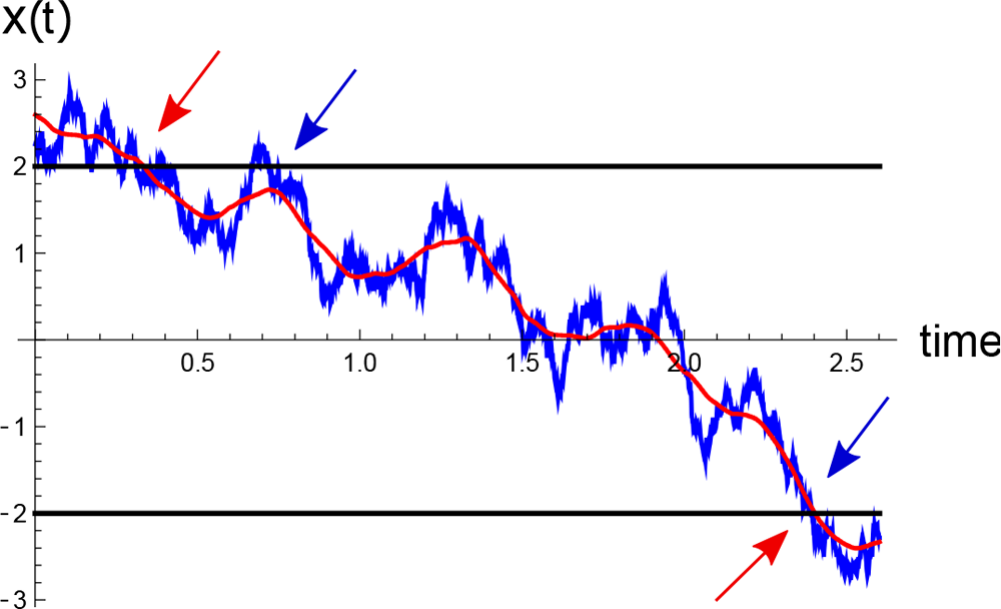
An example of a segment of a trajectory containing a transition path from *b* = +*L* to *a* = −*L* (these boundaries are indicated as horizontal lines). The simulated trajectory *x*(*t*) is shown in blue, and its smoothed version *x̃*(*t*) is in red. Smoothing was performed using eq 19 with Δ*t* ≈ 0.14 ⟨*t*_TP_⟩. The reduced transition path barrier height is *u* = 2. Blue/red arrows roughly indicate the beginning (i.e., entrance to the transition region (−*L*, *L*) and the end (exit from the transition region) of the transition path in the simulated/smoothed trajectories. The smoothed transition path has a longer temporal duration than the “true” one, with the true trajectory, unlike its smoothed counterpart, recrossing the boundary *b* multiple times.

An example of a transition path obtained for the smoothed trajectory *x̃*(*t*) and compared to the corresponding transition path for the unsmoothed trajectory *x*(*t*) is shown in Figure 5. Consistent with the discussion of Section 2, the transition path time for the smoothed trajectory is longer. In the particular example shown in Figure 5, the origin of this lengthening is clear: Smoothing eliminates some of the recrossings of the boundary where the transition path starts; the true transition path starts when this boundary is crossed for the last time (blue arrow in Figure 5), while the transition path obtained from the smoothed trajectory starts earlier (red arrow in Figure 5). We show below that this finding is general: the mean apparent transition path time derived from a smoothed trajectory *x̃*(*t*) is always longer than the true one.

### 3.1 Motional Averaging and Modified Potential of Mean Force

**Figure 6:**
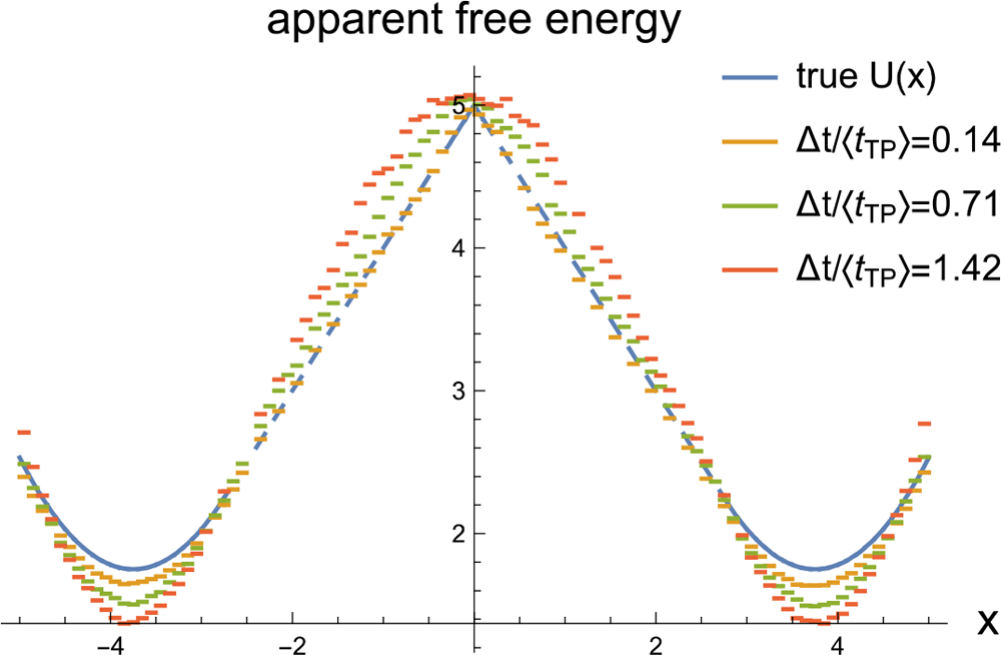
Apparent potential of mean force measured in *k*_B_*T* units (obtained via Boltzmann inversion of the histogram of the smoothed variable *x̃*, eq 19) plotted for different values of the smoothing time Δ*t*, as specified in the legend. The true potential here is the piecewise potential of Figure 2, with a cusp-shaped barrier and parabolic potential wells.

In general, the equilibrium probability distribution *p̃*_eq_(*x̃*) of the observed coordinate *x̃* is different from the distribution *p*_eq_(*x*) of the true value of *x*. As a result, application of the Boltzmann formula *p̃*_eq_(*x̃*) = *e*^–*βU*_app_(*x̃*)^ results in an apparent potential of mean force *U*_app_(*x*) that differs from the true one *U*(*x*).^28,29^ As seen from Figure 6, smoothing effectively deepens the potential wells of the apparent potential *U*_app_(*x̃*), increasing the apparent barrier. The origin of this effect is the “motional narrowing” that can be understood from the following argument: Imagine that the smoothing time Δ*t* is much longer than the relaxation time within a potential well. Then for a trajectory localized in one of the wells the time average of eq 19 will result in *x̃* having a very sharp distribution localized around the mean position within this well.^29^ For a trajectory undergoing transitions between the two wells, the time averaging will yield two such sharp peaks in the distribution of *p̃*(*x̃*) provided that the smoothing time Δ*t* is much shorter than the time scale for the transitions between the two wells. As the smoothing sharpens the two peaks of *p̃*(*x̃*) corresponding to each well and effectively reduces the apparent probability density *p̃*(*x̃* ≈ 0) of finding the system near the barrier top, it increases the apparent barrier between the two states.^28^

Importantly, Figure 6 shows that, even when the averaging time Δ*t* exceeds the mean transition path time, the effect of smoothing on the shape of the apparent potential of mean force is modest, and for Δ*t* ≪ ⟨*t*_TP_⟩ this effect is insignificant for the potential *U*(*x*) studied.

### 3.2 Mean Apparent Transition Path Time as a Function of the Smoothing Window

**Figure 7:**
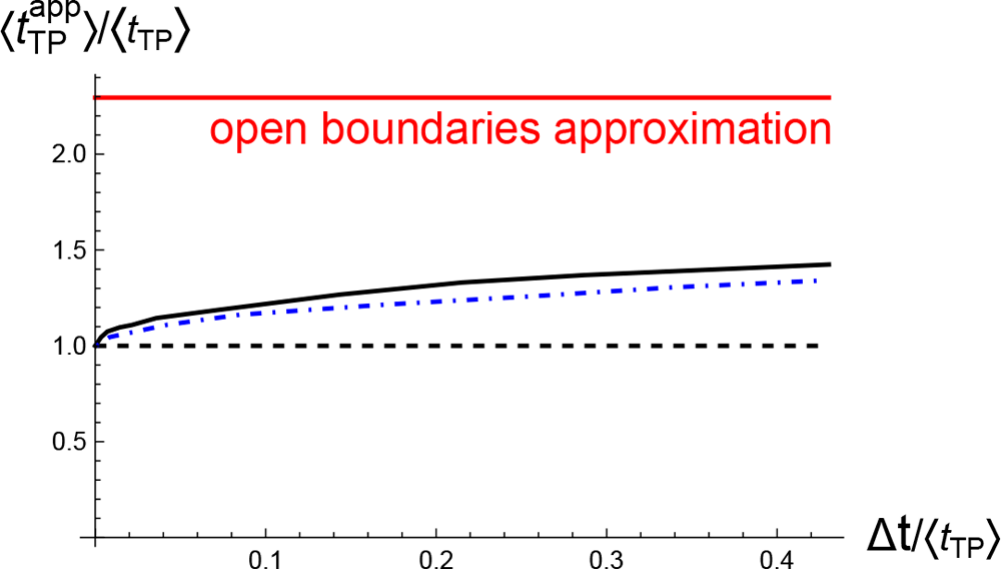
Apparent mean transition path time measured from smoothed Langevin trajectories, eq 19, as a function of the smoothing time window Δ*t*. Solid black line shows results for the (cusped) potential of Figure 2, and dot-dashed blue line shows results for a quartic double-well potential of the same depth and transition region boundaries chosen such that the reduced transition path barrier height is the same (*u* = 2). The horizontal dashed line indicates the true transition path time, and the horizontal solid (red) line shows the prediction of eq 17.

In accord with the discussion above, the apparent mean value of the transition path time ⟨*t*_TP_^app^⟩ based on the smoothed trajectory (eq 19) is longer than the true value, and it increases with increasing smoothing time window Δ*t* (Figure 7). The magnitude of the effect is relatively insensitive to the specifics of the potential, as seen in Figure 7, where the results are plotted both for the piecewise potential of Figure 2 and for a quartic double-well potential with the same depth and transition path barrier height.

It is reasonable to assume that an experimental technique that has its goal to measure the transition path times should operate in the regime Δ*t* < ⟨*t*_TP_⟩. As observed in Figure 6, the errors introduced by smoothing into the apparent potential of mean force in the barrier region −*L* < *x* < *L* are rather small in this regime, yet the increase in the apparent mean transition path time is significant (e.g., a ∼20% increase when Δ*t* ≈ 0.1⟨*t*_TP_⟩). At the same time, the apparent mean transition path time in this regime is significantly shorter than the value ⟨*t*_TP_⟩_open_ estimated using open boundary conditions and the comparable value of the mean conditional first passage time ⟨*t*_FP_^(*c*)^⟩ (see Figure 3). The latter two times, in a sense, represent the worst-case scenario where the experimental method fails to capture boundary recrossings.

## 4 Concluding Remarks

In this work, we studied the properties of transition-path trajectories and used them to test and understand various aspects that might affect measured transition paths and their interpretation. A key observation that underlies this work is that identification of the precise moment of time of boundary crossing signifying the beginning or the end of a transition path is beyond the resolution of current experiments, although it may be possible in the future. This limitation may substantially affect the interpretation of the measured transition path times. We note that the same limitation has to be considered when measuring other properties of barrier crossing dynamics such as transition path velocities or shapes.^11,13,30^

We have shown here that, as a result of limited time resolution, measured apparent transition path times are, on the average, always longer than the true ones. This effect is prominent with low barriers but becomes less so as the transition path barrier height increases.

When the observed transition paths can be viewed as smoothed versions of the true trajectories, the apparent transition path time increases as the smoothing time window increases. The changes in measured properties due to smoothing are significant even when the smoothing time is a small fraction (e.g., 10%) of the mean transition path time and the distortion of the apparent potential of mean force caused by smoothing is negligible. The effect of finite time resolution may be even more complicated when the experimental analysis involves additional data processing steps such as the maximum likelihood/hidden Markov analyses often used in single-molecule FRET experiments.^2,3,6,8,23,31−35^

In summary, the interpretation of folding/unfolding transition paths is challenging both from the point of view of the measurements and from the point of view of their analysis. We focused here on the latter. An interplay of experiment and theory is essential for interpreting experimental observations and deriving from them information, such as free energy profiles in the transition region. The combination of theoretical and experimental analysis is essential for making further progress in this stimulating field of study.

## Appendix A. Conditional First Passage Times in the Cusp-Shaped Potential Model

Here we consider a particle moving under the influence of a cusp-shaped potential of the form *U*(*x*) = −*F*|*x*|. The conditional first passage time from a boundary *a* = −*L* to *b* = +*L* can be calculated using eq 11 in the main text. To do so, we need the system’s Green’s function *G*(*x*, *t*| −*L*) satisfying the Smoluchowsky equation (eq 1)

A1

with the initial condition

A2

and absorbing boundary condition at *x* = *b* = *L*.

A3

Introducing the Laplace transform of this function

A4

eqs A1 and A2 lead to

A5

Using the ansatz

A6

one finds that the function *g* satisfies the equation

A7

whose general solution is of the form

A8

with

A9

This gives the general expression for the Laplace transform of the Green’s function.

A10

The values of the coefficients *A* and *B* are different in three distinct regions, *x* < −*L*, −*L* < *x* < 0, and *x* > 0. For *x* < 0, the coefficient *B* must vanish, as otherwise eq 10 would result in an unphysical solution that grows exponentially as *x* → −∞. The remaining coefficients can be obtained via matching of the solutions at the boundaries between these regions and using the boundary condition. Specifically, continuity requires that

A11

The presence of the delta function in eq A5 leads to discontinuity of the first derivative of *Ĝ* at *x* = −*L*. Indeed, integrating eq A5 from −*L* – 0 to −*L* + 0 one finds

A12

from which it follows that

A13

In contrast, the derivative is continuous at *x* = 0.

A14

Finally, *Ĝ* must satisfy the absorbing boundary condition (eq A3), which requires

A15

By solving eqs A11–A15 we thus obtain the Laplace transform of the Green’s function for all values of *x*. This solution is then used to obtain the Laplace transform of the distribution of the conditional first passage time (eq 11)

A16

and the Laplace transform of the flux at *x* = *L*, which is given by

A17

The final result for this flux can be written in the form

A18

The mean conditional first passage time can now be obtained using eqs 12 and A18.

A19

The resulting formula is rather long and will not be spelled out explicitly here.

## Appendix B. Transition Path Times Estimated Using Open Boundary Conditions for the Cusped-Shaped Potential Model

To compute the mean transition path time using open boundary conditions, one needs the function *G*_open_(*x*, *t*|*x*_0_). The Laplace transform of this function, *Ĝ*_open_, satisfies eqs A1 and A2 and can be found using the same approach as in Appendix A. The only difference is that the absorbing boundary condition, eq A15, must now be removed and replaced by the requirement that *Ĝ*_open_ does not diverge as *x* → ∞, which means that only the second, exponentially decaying term of eq A10 should be kept for *x* > *L*. The final result for the Laplace transform of the distribution of the transition path times approximated using open boundary conditions is

B1

with γ defined in eqs A9 and A18. Taking the inverse Laplace transform of this expression gives the distribution

B2

from which the mean time of the distribution, eq 17, is calculated.
